# Management of Edentulous and Atrophic Mandibular Fractures: A Systematic Review of Treatment Modalities and Outcomes

**DOI:** 10.7759/cureus.85164

**Published:** 2025-06-01

**Authors:** Munish Kumar, Ankush Gupta, Ajay Mittal, Harmandeep Kaur, Alisha Verma, Rishabh Kasrija, Seema Gupta

**Affiliations:** 1 Department of Oral and Maxillofacial Surgery, Guru Nanak Dev Dental College and Research Institute, Patiala, IND; 2 Department of Oral and Maxillofacial Surgery, Jagadguru Sri Shivarathreeshwara Dental College, Mysore, IND; 3 Department of Orthodontics, Kothiwal Dental College and Research Centre, Moradabad, IND

**Keywords:** atrophic, edentulous, fixation, fractures, management, mandible

## Abstract

Fracture of the edentulous mandible presents a significant clinical challenge due to anatomical alterations, reduced bone density, and lack of natural occlusal support, particularly in elderly patients with comorbidities. This systematic review aimed to evaluate and synthesize the available evidence on the treatment of edentulous and atrophic mandibular fractures, focusing on clinical outcomes, treatment success, and associated complications. A comprehensive search of four electronic databases was conducted to identify studies published between 1995 and 2024. Studies were selected based on predefined eligibility criteria following the Preferred Reporting Items for Systematic Reviews and Meta-Analyses (PRISMA) guidelines. Eligible studies included adult patients with edentulous mandibles who sustained mandibular fractures and were managed conservatively or surgically. Eight studies, involving 382 patients and at least 366 fracture sites, were included in the final analysis. Most studies have focused on atrophic mandibular fractures, with a few evaluating isolated body or condylar fractures. The most commonly used treatment approach was open reduction and internal fixation using locking reconstruction plates. Other modalities included compression plating, miniplate osteosynthesis with intermaxillary fixation, and conservative management in selected cases. Surgical access varied between intraoral and extraoral routes, with the latter providing improved outcomes in severely atrophic cases. Across studies, treatment outcomes were favorable, with bone union rates ranging from 74% to 100%. Complications, including soft tissue infections and transient nerve weakness, were infrequent and primarily minor. Non-union and reoperation rates were low. Despite the generally positive outcomes, the quality of evidence was limited. The risk of bias was rated as serious in seven out of eight studies due to confounding, missing data, and concerns regarding outcome measurement. These findings suggested that locking reconstruction plates offer reliable fixation and high success rates in managing edentulous and atrophic mandibular fractures. However, the overall evidence was constrained by the methodological limitations. Future research should focus on prospective studies with standardized protocols and long-term functional outcome assessments to enhance clinical decision-making in this complex patient population.

## Introduction and background

Jaw fractures in edentulous patients pose a distinct clinical challenge owing to the unique anatomical and physiological characteristics of this population [1]. The absence of teeth eliminates natural occlusal support, which is essential for conventional fracture reduction and stabilization techniques [2]. Compounding this, edentulous patients, often elderly, commonly exhibit atrophic jawbones, diminished bone quality, multiple comorbidities, increased fracture susceptibility, and complicated treatment outcomes [2]. The mandible, particularly when atrophic, is most frequently affected by progressive resorption after tooth loss, making it vulnerable to fractures caused by minimal trauma [1]. These fractures are difficult to manage because traditional dental occlusion-based fixation methods are not feasible, necessitating alternative approaches such as bone plating, Gunning splints, or prosthetic aids [2].

Management of jaw fractures in edentulous patients has evolved significantly over time. Early conservative approaches, such as external fixation and splints, often lead to prolonged healing and suboptimal results [3]. Modern surgical techniques, including rigid internal fixation with miniplates, locking plates, and reconstruction plates, have improved stability and recovery times [4]. However, there remains no standardized treatment protocol, as decisions are influenced by factors such as the extent of bone atrophy, fracture location (e.g., body, symphysis, or condyle), patient comorbidities, and available surgical expertise [5]. The choice between conservative and surgical interventions is complex, with each carrying a risk of complications such as non-union, infection, or hardware failure, particularly in frail elderly patients.

The existing literature on this topic is largely limited to retrospective studies, small case series, and narrative reviews, with a notable lack of high-quality prospective data [3-5]. This scarcity of robust evidence contributes to the absence of a consensus on optimal treatment strategies, forcing clinicians to rely on anecdotal experience or outdated protocols. Thus, a systematic synthesis of the available evidence is essential to provide clarity and guide evidence-based clinical decision-making for this challenging patient cohort.

This systematic review aimed to evaluate the existing literature on the management of jaw fractures in edentulous patients to identify effective treatment strategies and inform evidence-based clinical practices. By synthesizing clinical findings from the historical and contemporary literature, this review intends to provide a comprehensive reference for maxillofacial surgeons and researchers dealing with this complex patient group.

## Review

Methodology

**Figure 1:**
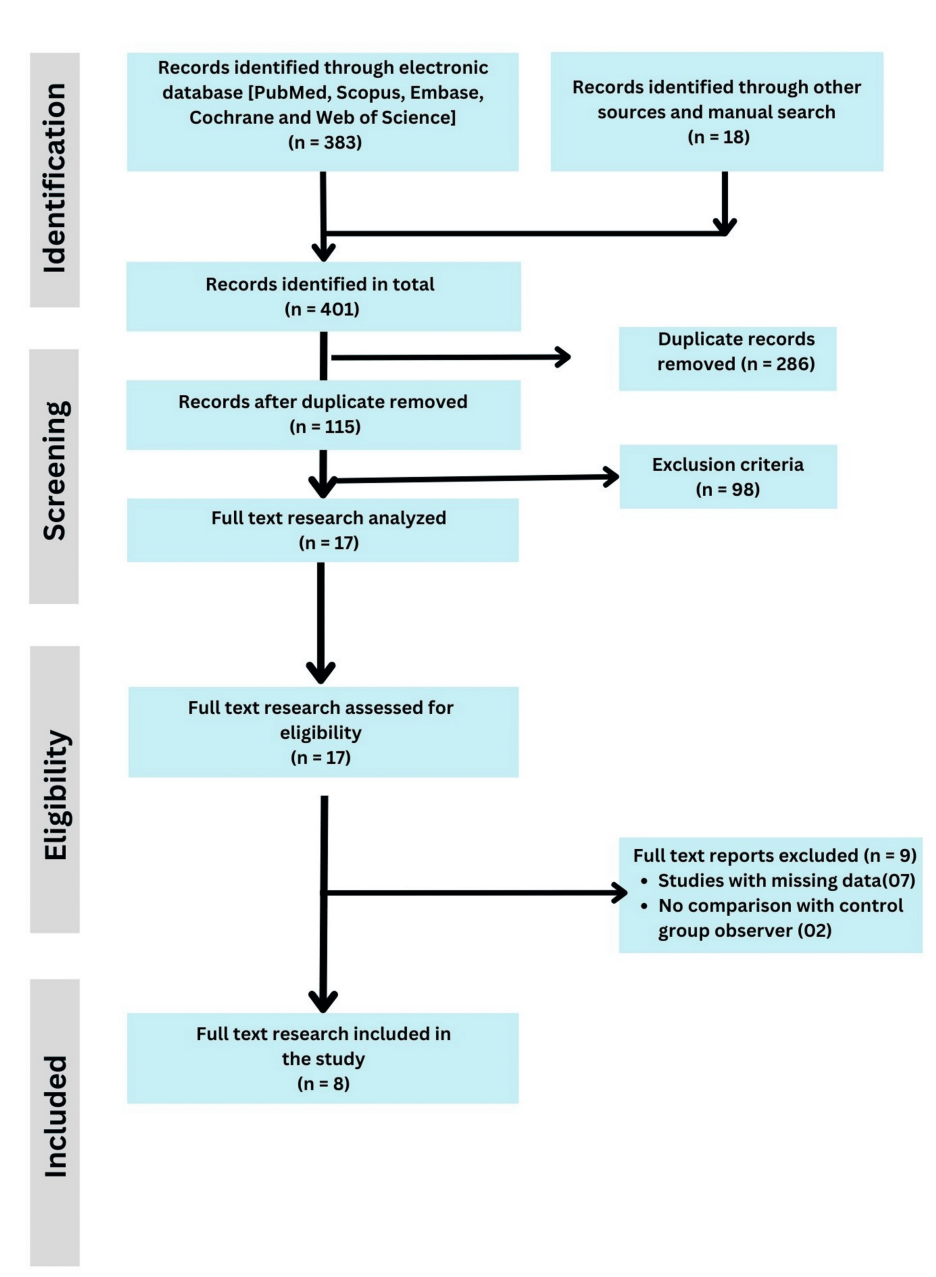
PRISMA flow chart. PRISMA: Preferred Reporting Items for Systematic Reviews and Meta-Analyses

This systematic review was conducted in strict accordance with the Preferred Reporting Items for Systematic Reviews and Meta-Analyses (PRISMA) guidelines to ensure transparent and robust methodology (Figure 1).

This review aimed to synthesize the evidence on the management of mandibular jaw fractures in edentulous patients, focusing on treatment modalities, clinical outcomes, and associated complications. Given the anticipated heterogeneity in study designs, patient populations, and reported outcomes, a qualitative narrative synthesis was planned, as a meta-analysis was expected to be infeasible.

The review was structured using the PICOS (Population, Intervention, Comparison, Outcome, Study Design) framework to clearly define eligibility criteria. The study population included adult patients aged 18 years or older with fully edentulous jaws who sustained mandibular fractures. Interventions encompassed any management strategy for jaw fractures, including conservative approaches such as external fixation, and surgical interventions such as open reduction and internal fixation (ORIF) with miniplates, locking plates, or reconstruction plates. Comparisons were made between different treatment modalities, such as specific fixation techniques, where data were allowed. Primary outcomes included treatment success, defined as fracture union and functional recovery, and complications, such as non-union, infection, or hardware failure. Secondary outcomes, where reported, included patient satisfaction. Eligible study designs were restricted to retrospective and prospective observational studies, such as cohort studies. Studies involving only dentate patients, those focused solely on non-jaw-related trauma, review articles, technical notes, case reports, case series, editorials, expert opinions without patient data, animal studies, and studies not published in English were excluded.

A comprehensive literature search was conducted across PubMed, Scopus, Web of Science, and the Cochrane Library to identify relevant studies published from 1 January 1995 to 31 December 2024. The search strategy, developed in collaboration with a medical librarian, combined Medical Subject Headings (MeSH) terms and free-text keywords, including “edentulous mandible”, “atrophic mandible”, “edentulous mandibular jaw”, “mandibular fracture”, “fracture management”, “fracture treatment”, “internal fixation”, and “external fixation”. Boolean operators (AND, OR) were employed to refine the search, with filters applied to limit the results to human studies and English language publications. Manual screening of reference lists from the included studies and key maxillofacial surgery journals was performed to identify additional relevant studies not captured in the electronic search.

Two independent reviewers conducted the study selection process and initially screened titles and abstracts against the eligibility criteria. Full-text articles of potentially relevant studies were retrieved and assessed for inclusion. Disagreements between the reviewers were resolved through discussion, and if consensus could not be reached, a third reviewer was consulted to make the final decision. This rigorous process minimized selection bias, and a PRISMA flow diagram was used to document the number of studies screened, included, and excluded, along with the reasons for exclusion (Figure 1).

Data extraction was performed using a standardized form to capture key study characteristics and outcomes, including author(s), year of publication, study design, sample size, fracture location (specifying sites such as the condyle, body, or symphysis), treatment modality (detailing techniques such as miniplates, locking plates, or external fixation), fixation technique specifics, primary and secondary outcomes, complications, and follow-up duration. Two reviewers independently extracted data, and discrepancies were resolved through discussion or consultation with a third reviewer to ensure accuracy and consistency.

Because of the predominance of retrospective and prospective observational studies, standard risk of bias tools, such as the Risk of Bias in Non-randomized Studies of Interventions, Version 2 (ROBINS-I V2) assessment tool, as given in November 2024, were applied, with adaptations for observational studies. The assessment evaluated domains such as selection bias, confounding, and completeness of outcome reporting. Methodological limitations, such as small sample sizes or lack of control groups, were qualitatively summarized to provide a critical evaluation of evidence quality. Covidence (Melbourne, Australia) for streamlined screening and data extraction and Rayyan (Qatar Computing Research Institute, Doha, Qatar) for efficient citation screening and deduplication were used. The overall body of evidence was rated as limited due to methodological constraints, with seven out of eight studies having a serious risk of bias, primarily from confounding and incomplete data, and only one study was rated with a moderate risk of bias.

The systematic review highlighted significant heterogeneity among the eight included studies, which complicated direct comparisons and precluded meta-analysis. This heterogeneity stemmed from variations in study designs (primarily retrospective and prospective observational), patient populations (differing ages, comorbidities, and degrees of mandibular atrophy), fracture types (e.g., body, condylar, or symphysis), and treatment modalities (e.g., locking reconstruction plates, miniplate osteosynthesis, compression plating, or conservative management). Surgical approaches also varied, with intraoral and extraoral routes applied inconsistently. Outcome reporting was inconsistent, with differences in follow-up duration, definitions of success (e.g., bony union rates), and complication assessments. These factors, combined with methodological limitations such as small sample sizes and lack of standardized protocols, contributed to the heterogeneity, limiting the ability to draw definitive conclusions and emphasizing the need for standardized, prospective studies.

Results

Eight studies were included in this review [6-13]. Details of the included studies are presented in Table 1. Eight studies involving 382 patients with fractures of the edentulous or atrophic mandibles were included in this systematic review. The majority of cases involved atrophic mandibular fractures, which were the primary focus in six studies [6-10,13]. Two studies addressed unilateral fractures in specific regions, with one focusing on the mandibular body [11] and the other on condylar fractures [12]. Overall, at least 366 fracture sites were documented, although not all the studies provided precise fracture counts. Among the available data, Luhr et al. [6] reported 84 fractures, Iatrou et al. [8] on 67 sites, Wittwer et al. [9] on 40 fractures, and Stathopoulos et al. [13] on 48 fractures.

**Table 1:** Summary of included studies.

S. No	Author (Year of publication)	Study type	Patients	Site/Focus	Treatment modalities	Outcome/Conclusion/Complication
1..	Luhr et al. 1996) [6]	Retrospective	84 fractures	Mandibular atrophic fractures	Compression plating	81 (96.5%) of the 84 fractures an uncomplicated, solid, bony union was achieved. Minor soft tissue infections, without interference with fracture healing, were observed in 6 (7%) cases
2.	Eyrich et al. (1997) [7]	Retrospective study	34 patients	Edentulous mandibular fractures	Different plating techniques	Favorable outcomes with internal fixation. 25 (74%) patients showed good bony union
3.	Iatrou et al. (1998) [8]	Prospective clinical study	40 patients with 67 fracture sites.	Edentulous mandibular fractures	Intraoral approach with intermaxillary fixation, and miniplate osteosynthesis	Safe and effective in elderly patients with reoperation rate of only 2.9% (2 fractures)
4.	Wittwer et al. (2006) [9]	Retrospective study	40 fractures	Atrophic mandibular fractures	Open reduction and internal fixation	Uneventful healing in 36 (90%) fractures, non-union in 4 (10%) fractures. Minor complications like infections in 6 (15%) fractures
5.	Gerbino et al. (2018) [10]	Multicenter retrospective	55 patients	Atrophic mandibular fractures	External open reduction and rigid fixation (ORIF) with locking, load-bearing plates	Adequacy of reduction was good in most of the patients. Transient weakness of the marginal branch of the facial nerve was recorded in 11 (20%) patients and permanent weakness in 2 (36%) patients. All patients achieved a complete fracture healing
6.	Brucoli et al. (2020) [11]	Retrospective study	43 patients	Unilateral body fractures of edentulous atrophic mandible	Locking reconstruction plates	Effective fixation with low complication rates. Complication in 13 (30%) cases. All patients were satisfied
7.	Brucoli et al. (2020) [12]	Multicenter retrospective	52 patients	Unilateral condylar fractures in atrophic mandibles	No treatment was performed in 37 cases, closed reduction in 4 patients, and 11 patients underwent open reduction and internal fixation	Open reduction was preferable in severe atrophy. 48 (92%) patients were satisfied with no complications
8.	Stathopoulos et al. (2024) [13]	Prospective	34 patients with 48 fractures	Atrophic mandibular fractures	Locking reconstruction plate	Excellent results if used with external approach

Various treatment modalities were utilized across studies, with ORIF being the most commonly employed approach. Locking reconstruction plates featured prominently in four studies [10-13] and were often used in conjunction with either intraoral or extraoral surgical access. One study [6] reported the use of compression plating, while another [8] employed miniplate osteosynthesis with intermaxillary fixation via an intraoral approach. Mixed plating techniques were described by Eyrich et al. [7], and conservative management, including closed reduction or no intervention, was reserved for condylar fractures in cases with minimal symptoms [12].

The overall clinical outcomes were favorable, with consistently high rates of fracture healing and patient satisfaction. Reported bony union success rates range from 74% to 100%. Luhr et al. [6] achieved a 96.5% rate of solid bony union with compression plating, whereas Eyrich et al. [7] reported a 74% success rate using various internal fixation techniques. In a study by Wittwer et al. [9], uneventful healing occurred in 36 (90%) cases, whereas Gerbino et al. [10] and Stathopoulos et al. [13] documented complete fracture healing in all patients treated with locking plates and rigid fixation. Brucoli et al. [11,12] also reported successful outcomes, with all patients expressing satisfaction regardless of whether they underwent surgical or conservative management.

The complication rates were generally low and mostly minor. Infection was the most frequently observed complication, occurring in six (7%) patients in the study by Luhr et al. [6], in six (15%) cases in Wittwer et al. [9], and in 13 (30%) cases reported by Brucoli et al. [11]. Gerbino et al. [10] observed transient weakness of the marginal mandibular nerve in 11 (20%) patients and permanent weakness in two, although all patients ultimately achieved complete bone healing. Non-union was relatively uncommon, reported in 4 (10%) fractures by Wittwer et al. [9], and the need for reoperation was minimal, noted in only 2 (2.9%) cases in Iatrou et al.’s prospective study [8].

Regarding the surgical approach, the extraoral route appeared to enhance outcomes when used in combination with locking plates, as emphasized by Stathopoulos et al. [13], who reported excellent results with this method. In contrast, the intraoral approach employed by Iatrou et al. [8] was safe and effective, particularly in elderly patients, and was associated with minimal need for retreatment.

The risk of bias among the included non-randomized studies was predominantly rated as serious, particularly owing to confounding factors. Seven of the eight studies were judged to have a serious risk of bias in this domain, likely reflecting the inherent limitations of retrospective or observational designs, where potential confounders may not have been adequately controlled [6-8,10-13]. The classification of interventions and selection of participants into studies were consistently rated as low risk across all studies, indicating the appropriate identification and inclusion of study subjects. However, the risk due to missing data and outcome measurement was moderate in most studies and serious in four studies, potentially impacting the reliability of reported treatment effects [10-13]. Only one study was rated as having a moderate overall risk of bias [9], while all others were categorized as having a serious overall risk, primarily driven by issues related to confounding and incomplete data. These findings suggest that while the included studies provided valuable clinical insights, the quality of evidence was limited by methodological constraints, which should be considered when interpreting the outcomes (Table 2).

**Table 2:** Risk of bias assessment using the Risk of Bias In Non-randomized Studies – of Interventions, Version 2 (ROBINS-I V2) assessment tool.

S No.	Author with publication year	Risk of bias due to confounding	Risk of bias in classification of interventions	Risk of bias in selection of participants into the study	Risk of bias due to deviations from intended interventions	Risk of bias due to missing data	Risk of bias arising from measurement of the outcome	Risk of bias in selection of the reported result	Overall
1.	Eyrich et al. (1997) [6]	Serious	Low	Low	Low	Low	Moderate	Moderate	Serious
2.	Luhr et al. (1996) [7]	Serious	Low	Low	Low	Low	Moderate	Moderate	Serious
3.	Iatrou et al. (1998) [8]	Serious	Low	Low	Low	Low	Moderate	Moderate	Serious
4.	Wittwer et al. (2006) [9]	Moderate	Low	Low	Low	Low	Moderate	Moderate	Moderate
5.	Gerbino et al. (2018) [10]	Moderate	Low	Low	Low	Serious	Moderate	Moderate	Serious
6.	Brucoli et al. (2020) [11]	Serious	Low	Low	Low	Serious	Moderate	Moderate	Serious
7.	Brucoli et al. (2020) [12]	Serious	Low	Low	Low	Serious	Moderate	Moderate	Serious
8.	Stathopoulos et al. (2024) [13]	Serious	Low	Moderate	Low	Serious	Moderate	Moderate	Serious

Discussion

The management of edentulous and atrophic mandibular fractures presents a unique clinical challenge owing to diminished bone quantity and quality, altered biomechanics, and often compromised systemic health in elderly patients [3]. The findings of this systematic review, which included eight studies comprising 382 patients, offer valuable insights into the various surgical approaches and fixation techniques used in the treatment of such fractures. Despite the methodological limitations inherent to non-randomized studies, the overall outcomes suggest that modern fixation strategies, particularly the use of locking reconstruction plates and ORIF, are effective in promoting fracture healing and restoring mandibular function.

Atrophic mandibular fractures are common in geriatric patients with prolonged edentulism [2,3]. Reduced bone volume, impaired vascularity, and absence of teeth as natural stabilizers create a complex environment for fracture healing. In this review, the majority of studies focused on this subset of fractures, with a consistent trend toward the use of load-bearing fixation methods such as locking plates [14]. These devices are specifically designed to provide rigid stability, even in severely atrophic mandibles, where conventional miniplates may fail because of poor cortical engagement. According to a study by Harjani et al. [14], locking plates led to better stability to bear masticatory loads during the process of osteosynthesis, compared to non-locking plates. This bone-plate construct functions as an internal-external stabilizer, leading to an enhanced load distribution and reducing the likelihood of stress concentration on a singular screw, thereby mitigating the potential for screw loosening and stripping. Furthermore, as precise anatomical conformity of the plate to the underlying bone topology is not imperative, there are theoretically fewer disruptions to the neighboring vascular supply [15,16].

Data synthesis indicated a high overall success rate across treatment modalities, with fracture healing observed in 90-100% of cases in most studies. Notably, Luhr et al. [6] and Gerbino et al. [10] achieved near-universal healing outcomes using compression and locking reconstruction plates, respectively. Similar results have been reported in other studies [14,17]. Levine et al. [18] used an Eccentric Dynamic Compression Plate (EDCP) and a Dynamic Compression Plate (DCP) for mandibular fracture fixation and obtained good results. Similarly, Stathopoulos et al. [13] emphasized the benefit of combining locking plates with an extraoral surgical approach, which provides better access for optimal plate positioning and screw engagement. These results support the notion that rigid fixation is paramount for achieving stability in atrophic mandibles and for reducing the risk of non-union or malunion.

A segment of the existing literature posits that the intraoral technique is superior [8,19], whereas another advocates the extraoral approach [13,20]. The intraoral pathway mitigates the potential risk of compromising the marginal branch of the facial nerve and eliminating the presence of an external scar, thereby facilitating single-layer mucosal closure. Conversely, the external approach guarantees sufficient exposure of the fragments, leading to a more meticulous surgical process (notably enabling adaptation of a larger profile plate) [13]. Consequently, the external method diminishes the likelihood of contamination by oral microorganisms and safeguards against damage to the inferior alveolar bundle, which typically resides atop the alveolar crest in atrophic mandibles [13]. Similar results were reported by De Feudis et al. [21], who reported better results with rigid fixation and an extraoral approach for managing atrophic mandibles.

Iatrou et al. [8] reported a high success rate using an intraoral route with miniplate osteosynthesis, although their patient cohort was relatively younger and medically stable. In contrast, Brucoli et al. [12] found open reduction to be more favorable in cases of severe mandibular atrophy, particularly in condylar fractures, in which anatomical complexity and mechanical forces necessitate more stable fixation. Advancements in geriatric care, along with enhanced preoperative and postoperative protocols, indicate that ORIF, adhering to the principles of load-bearing, secures optimal osseous healing and facilitates the swift restoration of functional capabilities while circumventing the discomfort associated with prolonged inter-maxillary fixation. Therefore, this technique may be regarded as a benchmark for fractures of the atrophic mandible [21].

An important finding across studies was the low incidence of major complications. Infections have been reported in a few studies, such as those by Luhr et al. [6] and Wittwer et al. [9], but were generally superficial and did not interfere with fracture healing. Neurological complications were uncommon, with transient facial nerve weakness documented in the study by Gerbino et al. [10], which was likely attributable to the external surgical approach [13,21]. Despite these minor issues, the overall patient satisfaction was high in studies that reported subjective outcomes, reflecting both the functional and aesthetic success of the treatments.

These findings have significant clinical implications. First, locking plate systems should be considered the standard of care for atrophic mandibular fractures owing to their biomechanical superiority. Second, the choice between intraoral and extraoral approaches should be dictated by the fracture type, degree of atrophy, and the surgeon's experience. In cases of significant bone loss or unfavorable fracture patterns, an extraoral approach may yield more predictable outcomes. Third, patient-specific factors, including age, systemic comorbidities, and pre-injury functional status, must be carefully assessed when planning surgical interventions.

Despite the encouraging outcomes, this review was limited by the quality and design of the included studies. All eight studies were non-randomized, and most were retrospective in nature. According to the risk of bias assessment tool, seven studies were deemed to have a serious overall risk of bias, primarily due to confounding variables and incomplete data reporting. Only one study [9] was rated as having a moderate risk of bias. These methodological flaws restrict our ability to draw definitive conclusions or perform meaningful meta-analyses. Moreover, heterogeneity in fracture types, fixation methods, and surgical approaches further complicates direct comparisons across studies.

Another limitation is inconsistency in outcome reporting. While most studies reported healing rates and general complications, few included long-term functional outcomes, quality of life measures, or objective assessments of masticatory performance. There have also been limited reports on specific technical variables, such as plate design, screw length, or angulation, which are critical details that influence treatment success. The lack of standardized follow-up periods across studies also weakens the strength of the outcome evaluation.

Given these gaps, future research should prioritize prospective multicentre clinical trials with standardized protocols to evaluate the efficacy of different treatment modalities. Although challenging in this surgical context, randomized controlled trials (RCTs) provide high-level evidence necessary to guide treatment guidelines. Future studies should also aim to incorporate patient-centred outcomes, including pain levels, dietary function, prosthetic rehabilitation potential, and overall quality of life.

Advancements in technology offer additional avenues to improve care. Three-dimensional virtual surgical planning, patient-specific implants (PSIs), and navigation-assisted surgery are promising tools for enhancing the accuracy of fracture reduction and fixation, particularly in anatomically complex cases [22]. Further investigation is needed to assess the cost-effectiveness and clinical benefits of these technologies for treating atrophic mandibular fractures.

Additionally, the role of bone grafting and biological enhancers, such as bone morphogenetic proteins or platelet-rich fibrin, should be explored in patients with severe bone resorption or poor healing capacity [23-25]. These adjunctive therapies may accelerate bone regeneration and improve the outcomes in high-risk populations.

The current body of literature supports the use of ORIF with locking plate fixation as a reliable and effective strategy for managing edentulous and atrophic mandibular fractures [13,21]. Although surgical technique and hardware selection remain critical determinants of success, careful patient evaluation and appropriate surgical planning are equally essential. Despite methodological limitations in existing studies, cumulative evidence suggests favorable healing outcomes with minimal complications. To strengthen the evidence base, future research should focus on high-quality prospective studies, incorporate advanced technologies, and explore adjunct regenerative therapies. Such efforts will be vital for optimizing treatment strategies and improving the standard of care for this complex and growing patient population.

## Conclusions

This systematic review demonstrated that ORIF using locking reconstruction plates is the most effective and reliable approach for managing edentulous and atrophic mandibular fractures, offering high rates of bony union and low complication rates. Although both intraoral and extraoral approaches can be successful, the extraoral route may be more advantageous in cases of severe atrophy or complex fracture patterns. Despite favorable clinical outcomes, the overall quality of evidence is limited by the predominance of retrospective, nonrandomized studies with a serious risk of bias. High-quality prospective trials and standardized outcome measures are necessary to establish definitive treatment protocols and enhance clinical decision making in this challenging patient population.
